# Selection of massive bone allografts using shape-matching 3-dimensional registration

**DOI:** 10.3109/17453671003587127

**Published:** 2010-04-06

**Authors:** Laurent Paul, Pierre-Louis Docquier, Olivier Cartiaux, Olivier Cornu, Christian Delloye, Xavier Banse

**Affiliations:** ^1^Department of Orthopaedic Surgery, Saint-Luc University Hospital (UCL Université Catholique de Louvain), BrusselsBelgium; ^2^Center for Research in Mechatronics (CEREM), UCL Université Catholique de Louvain, Louvain-La-NeuveBelgium

## Abstract

Background and purpose Massive bone allografts are used when surgery causes large segmental defects. Shape-matching is the primary criterion for selection of an allograft. The current selection method, based on 2-dimensional template comparison, is inefficient for 3-dimensional complex bones. We have analyzed a 3-dimensional (3-D) registration method to match the anatomy of the allograft with that of the recipient.

Methods 3-D CT-based registration was performed to match the shapes of both bones. We used the registration to align the allograft volume onto the recipient's bone. Hemipelvic allograft selection was tested in 10 virtual recipients with a panel of 10 potential allografts, including one from the recipient himself (trap graft). 4 observers were asked to visually inspect the superposition of allograft over the recipient, to classify the allografts into 4 categories according to the matching of anatomic zones, and to select the 3 best matching allografts. The results obtained using the registration method were compared with those from a previous study on the template method.

Results Using the registration method, the observers systematically detected the trap graft. Selections of the 3 best matching allografts performed using registration and template methods were different. Selection of the 3 best matching allografts was improved by the registration method. Finally, reproducibility of the selection was improved when using the registration method.

Interpretation 3-D CT registration provides more useful information than the template method but the final decision lies with the surgeon, who should select the optimal allograft according to his or her own preferences and the needs of the recipient.

## Introduction

**Figure 1.**
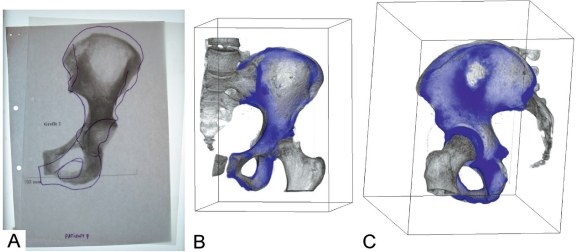
A. An example of the template method. It consists of a comparison between a fluoroscopy and a manually drawn template from an anteroposterior radiography of the recipient. B and C. Example of registration method (3-D views). The user can rotate the 3-D view to examine bone congruency. 2-D slices can also be inspected.

Selection of the best massive bone allograft to match a host bone remains a problem (Muscolo et al. 2006, Delloye et al. 2007). The commonest selection method relies on the comparison of allograft fluoroscopies (or contact radiographs) with the anteroposterior radiograph (or template) of the recipient (Figure 1A) (Delloye et al. 1991, Virolainen et al. 2003, Meehan et al. 2005). However, this “template” selection method does not allow the selection of an optimal hemipelvic allograft (Paul et al. 2008). The complex osseous architecture of pelvic allografts especially requires the use of shape-matched allografts (Delloye et al. 2007). A better method is required, certainly for pelvic transplants but also for any allograft where shape matching is important.

We investigated a new selection method based on 3-D registration of CT scans from the recipient and the allograft. We studied (1) whether the registration method would be able to identify an allograft that perfectly matches the recipient, (2) whether it would produce a selection of best-matching allografts similar to that obtained with the conventional template method, and (3) whether it permits a more reproducible allograft selection than the template method.

## Material and methods

### Experimental set-up

We compared bone allograft selection using the template and registration methods. We used the same framework as in our previous experiment on the template method (Paul et al. 2008). In that study, 4 observers performed allograft selection for 10 virtual recipients from a panel of 9 real allografts and 1 trap graft. For each recipient, a trap graft was created using the recipient's own hemipelvis. Observers were provided with the shape of recipient's bone drawn on tracing paper (template) and a planar image of the allograft. These images formed the basis of the selection process. Observers were asked to position the template over the allograft in such a way as to best superimpose the sacroiliac and pubic joints (Figure 1A). This method is the current procedure for bone allograft selection at our institution. In a clinical situation, the allograft is then delivered to the operating surgeon. In both the previous (template) study and the present (registration) study, 2 surgeons and 2 allograft coordinators were asked to categorize allografts according to how well they matched the recipient. After optimal positioning of the template over the allograft image, observers visually checked 4 criteria: congruency of 2 joints (sacroiliac and pubic joints), and 2 distances (iliac wing width and pelvic height). To check the congruency of joints, observers measured the overlapping area of articular surfaces with a ruler. At least 50% of the area of the joint must be superimposed to validate sacroiliac or pubic congruency, which are the 2 most important criteria to validate since they are crucial to the reconstruction. This is the rationale for ascribing them a weighting of 2. To check distance criteria, observers measured the difference in height or width between anteroposterior images of the allograft and recipient. The distance criterion was validated when this difference was less than 10 mm. The weighting of distance criteria was 1, since an error in these distances is considered to be less important for the reconstruction. After scoring, each allograft for each recipient was classified into 4 categories: adequate (6 points, all criteria validated), acceptable (5 points, all but 1 distance criterion), inadequate (4 points, all criteria but 1 joint or 2 distances), or unacceptable (less than 4 points). Finally, the observers were asked to select the 3 best matching allografts for a given recipient among those identified as adequate or acceptable. Clinically, this final selection is the most relevant information.

**Figure 2.**
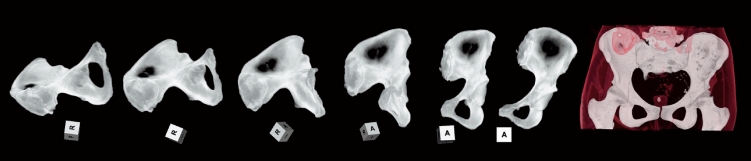
The overall principle of a registration algorithm. A moving volume (allograft on the left) is superposed over a fixed volume (recipient on the right) using an optimized spatial transform. The iterative characteristic of the process is illustrated by the gradual alignment toward the recipient.

**Figure 3.**
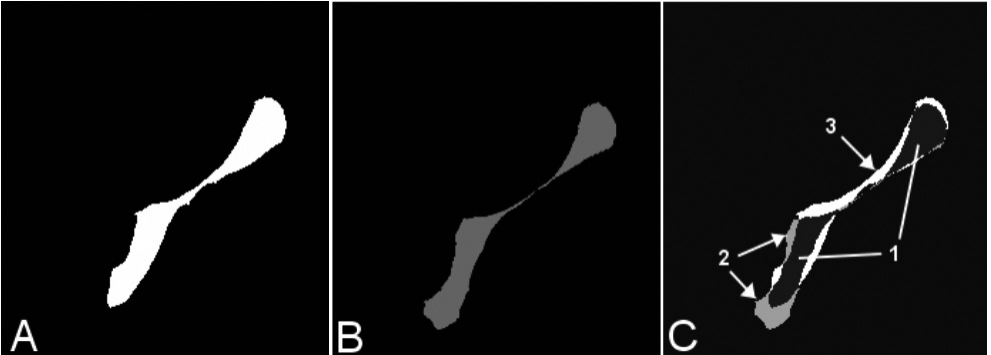
A–C. Transversal 2-D slices extracted from the binary recipient (A) and the binary allograft after registration (B). The term “binary” is related to the fact that only 2 kinds of voxels can be found in these volumes (“1” where bone is present, “0” for the background). At the end of the registration process, an overlay volume (C) is automatically produced merging the 2 binary volumes (A and B). Different gray values are attributed to the voxels in the overlay: dark gray where the allograft is overlapping the recipient (panel C, 1), light gray where the allograft is larger than the recipient (panel C, 2), and white where the recipient is larger than the allograft (panel C, 3).

**Figure 4.**
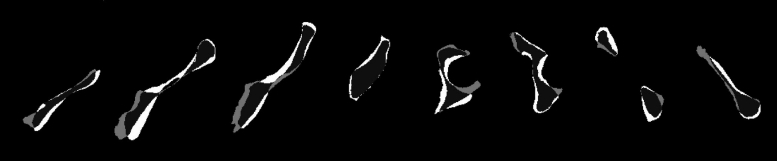
Eight transversal slices of an overlay volume are shown for recipient 7 and allograft 2. In the experiment, the overlay volume is compounded of approximately 200 2-D transverse slices. Observers had to traverse the volume using transversal and coronal slices and check 4 criteria visually.

In the present study, we developed and tested a new selection method that aims to align the allograft volume onto the recipient's bone (Figure 1B and C). An experiment was conducted using the same virtual recipients, allografts, and the trap grafts as used in the study on the template method. The trap graft was again present in the panel. We performed an iterative registration technique (Figure 2) on the 10 allografts with the 10 recipients to find the optimal alignment of each allograft over each recipient's bone. Then, an overlay volume was computed merging optimally aligned allograft and recipient (Figure 3C and Figure 4). It allows the observer to visualize joint congruency either in 3-D or 2-D slices extracted from this volume. To quantify allograft and recipient matching, observers were asked to check the same 4 criteria previously defined using 2-D and 3-D views provided by Volview (Version 2.0.5; Kitware Inc, New York, NY). Overall congruency of joints was evaluated, scanning all 2-D slices that showed the joint to estimate the overlapping of allograft over recipient. If the observer considered that at least 50% of the recipient's junction was overlapped by the allograft, then the criterion was validated. Distance criteria were checked by measuring the difference in height or width between allograft and recipient using a 2-D measuring tool provided by Volview. Again, when the difference was lower than 10 mm, the criterion was validated. After scoring, allografts were classified into the same 4 categories as in the template experiment. Finally, observers also selected the 3 best-matching allografts for each recipient among those identified as adequate or acceptable.

### Image preprocessing

**Figure 5.**
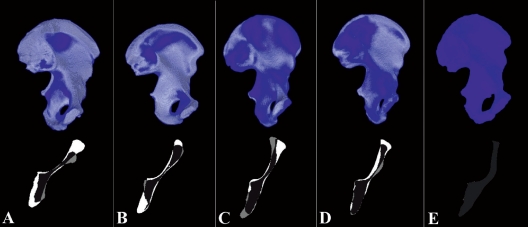
Five examples of overlay volumes from a poor recipient/allograft match (categorized as unacceptable) (A) to a good match (categorized as adequate) (D). Panel E shows a perfect match of the recipient with the trap (after alignment by registration). The top represents a 3-D view of the overlay volume with 2 colors: recipient in light blue, and graft in darker blue. [Authors: OK?] The bottom shows slices extracted from the same overlay volume at the level of the sacroiliac joint. Panel E shows the perfect alignment achieved by the registration method. On the corresponding 2-D slice, only 3 voxels were not overlapping. This is the systematic error produced by the registration method.

This experiment was performed using recipient and allograft CT scans that had been collected during the template experiment. All allografts had been scanned using standard acquisition parameters (1 mm spacing between slices, slice thickness 2.7 mm, 1.0 second per 360° rotation, peak 90 kV). Parameters of recipient CT scans were not standardized since they were acquired for common clinical indications. Nevertheless, a minimum spatial resolution was required (1 mm spacing between slices and slice thickness 2.7 mm). Allografts had been scanned frozen, wrapped in their sterile packing, and exposed for less than 5 min to ambient temperature. Recipient CT scans were retrieved from PACS (Picture Archiving and Communication System). All applications used for the experiment were developed using the Insight Toolkit (ITK) library of image analysis algorithms (Ibanez et al. 2003). Allograft and recipient CT scans were preprocessed. This preprocessing consisted of cropping, thresholding, and binarization. Volumes were cropped to a region of interest enclosing the pelvic bone. Thresholding was performed using a “threshold below” technique; voxels with gray values lower than 1,100 Hounsfield units were blackened. This step removes soft tissues from recipient and allograft volumes. Cropping and thresholding produce volumes that are more suitable for the registration, which is then focused on the target bone to reach the best possible alignment of the allograft over the recipient. Binarization of the allografts and the recipients was performed using open-source software, ITK-Snap 1.4.1 (Yushkevich et al. 2006) (www.itksnap.org). Voxels in which bone was present were set to “1” (white), whereas all others were set to “0” (black). These binary volume were used at the end of the registration process to produce overlay volumes (Figures 3C, 4, and 5).

To prevent any bias in the experiment, the trap graft must be handled as a real allograft. Thus, registration must also be performed between the trap graft and the recipient. They are perfectly aligned, however, since the volumes involved are identical. Registration cannot work on 2 identical volumes, since their alignment is already perfect. Thus, an initial rotation and translation (spatial transformation) was applied to the trap graft to let the registration work and to find an optimal alignment. This initial transformation of the trap graft simulates an arbitrary position in the CT scanner as if the trap was a real allograft.

### Registration algorithm

The goal of registration is to spatially align 2 images (2-D) or volumes (3-D) acquired using different devices or at different times. This multifaceted process can be rigid or deformable, intra- or intermodality, intra- or intersubject, and volume-, surface-, or point-based. The technique is widely used in the medical field, for example to build anatomic atlases (Thompson et al. 2000), to delimit tumors with images from different modalities (Nishioka et al. 2002), to monitor tumor growth (Haney et al. 2001), and to match a patient with his or her own images in computer-assisted surgery (Merloz et al. 1998). Multiple reviews have summarized the large body of work that has been done on registration (Maintz and Viergever 1998, Zitova and Flusser 2003). However, few studies have involved matching 2 different objects. Bone grafting requires such a method to provide a merged view of the host and graft bones. To meet our expectations, a rigid registration would be needed to preserve distances and to compare bone shapes without distortions. Rigid means that there are no scaling factors or distortions of the volumes involved in the registration. Distances are preserved in order not to distort the bones. In the process, one volume was considered “fixed” (recipient), whereas the second was considered to be “moving” (allograft) and underwent spatial transformation (Figure 2). For each iteration, a temporary moving volume is computed by applying the current spatial transform. A voxel's gray value from the fixed volume was compared with the corresponding voxel's gray value from the temporary moving volume. This produced a similarity measure called the metric value, defined as


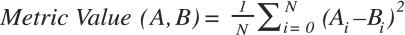


where *A_i_* is the i-th voxel of volume A (recipient), *B_i_* is the i-th voxel of volume B (allograft), and N is the number of voxels considered (usually 10^5^).

The translation and rotation parameters of the spatial transform were adapted according to the metric value in what is known as the optimization process (gradient descent optimizer with an adaptive learning rate). The moving volume was transformed using these new transformation parameters to create a new temporary moving volume that was then used during the next iteration. This iterative loop was repeated until the metric value was minimized, at which point both volumes were considered to be optimally aligned. The final spatial transform was defined, and the transformed allograft volumes were created (linear interpolation; Ibanez et al. 2003). An overlay volume was computed by merging the binary recipient (Figure 3A) and the transformed binary allograft (nearest-neighbor interpolation; Figure 3B). 3 distinct gray values were attributed to the voxels regarding bone presence (Figure 3C). The resulting overlay volumes (Figures 4 and 5) were provided to observers who were asked to visually check the 4 criteria for each allograft/recipient combination using transverse and coronal images from Volview. These observers were blind as to the identity of the trap. The visual inspection of the 4 criteria permitted classification of allograft/recipient combinations into 4 categories and selection of the 3 best-matching allografts for a given recipient.

### Statistics

We tested whether the selection of the trap graft as the best matching allograft was statistically improved using the registration method rather than template method. Fisher's exact test was used to detect a difference between both methods. Fisher's test was preferred to the Chi-squared test because the expected frequencies were below 5. For all tests, we used a significance level of 0.05.

Cohen's kappa (Cohen 1960) was used to measure the overall intraobserver agreement in the selection of the 3 best-matching allografts using the 2 methods. With this test, we assessed whether template and registration methods would yield a similar selection.

Overall reproducibility (interobserver agreement) of the selection using the registration method was estimated using Cohen's kappa (Reproducibility _Registration_). This value was compared to the reproducibility of the template method (Reproducibility _Template_). A Z-test (Congalton and Green 1998) was performed on Reproducibility _Template_ and Reproducibility _Registration_ to detect any substantial improvement using the registration method rather than template method. A priori, we established that we would reject the hypothesis that 2 kappas were equal if Z was greater than 1.96 (for a 95% confidence level test).

## Results

The mean computing time for the registration process was 4 min on a conventional personal computer. 100 registrations were successfully performed, and registration always succeeded in reaching an alignment (no failure). Trap grafts were perfectly aligned with the corresponding recipient by the registration method. 400 observations were made (4 observers classified 10 allografts for each of 10 recipients). Visual inspection of the 100 overlay volumes took approximately 3 h for each observer, who scanned overlay volumes, checked the 4 criteria, scored the recipient/allograft combinations, and selected what he considered to be the 3 best-matching allografts.

Using the registration method, observers detected that a trap graft was inserted in the allograft panel. The trap graft was systematically qualified as “adequate” and identified as the best-matching allograft in all cases (10 detections over 10 observations for each of the 4 observers). Fisher's test demonstrated an improvement (p < 0.001) in the detection of the trap graft using the registration method rather than the template method (where there were only 3 detections over 40 observations).

Cohen's kappa, measuring the overall intraobserver agreement in selection between the registration and template methods, was 0.04 (95% CI: –0.10 to 0.18); agreement in selection was no better than that expected by chance. The selection of the 3 best-matching allografts using both methods was different.

Reproducibility _Registration_ was 0.75 (95% CI: 0.68–0.81), higher than Reproducibility Template (0.39; 95% CI: 0.28–0.50). According to the Z-test, the reproducibility in selection was improved using registration rather than templates (Z-score = 5.4; > 1.96).

## Discussion

The successful incorporation of a massive bone allograft requires a shape-matched allograft to restore the kinematics of the joint and to minimize the risk of fracture or non-union. Our previous study on the current template selection method showed that it allowed selection of the trap graft as the best-matching allograft in only 3 of 40 cases. The reproducibility of the template method was found to be low. Our main question was whether the registration method would lead to the selection of the best-matching allograft. However, there is no gold standard to define what a best-matching allograft is. This depends on many factors such as tumor location, the needs of the recipient, and the type of reconstruction (e.g. associated with prosthesis). The only standard for the best-matching allograft is the trap graft, because it is the ideal allograft. This ideal graft was introduced into the allograft panel as the trap graft. Overlay volumes produced by the registration allowed observers to always select this graft as the best-matching allograft. This result was expected, since the matching is performed by the computer. Observers could not miss its selection, and they could not miss an optimal allograft if there was one in the panel. Using the template method, the trap graft was rejected in 30 out of 40 cases (categorized as “inadequate” or “unacceptable”). The registration method improves the selection of the trap graft compared with the template method.

A second question was whether, in a clinical situation, the template and registration methods would lead to the same selection of 3 allografts for a given recipient. Our findings showed that observers do not select the same 3 allografts using the registration method and the template method. Since the template method did not permit detection of the perfect implant (trap graft), we state that the registration method is the best procedure available for selection of the optimal allograft. A third question was whether allograft selection using registration is more reproducible than when using the template method. We found that it was.

3 forms of bias explain the gross failure of the template method. Radiographic magnification induced by radiographs is the major one. Secondly, the lack of standard positioning of the recipient and allograft during image acquisition often leads to large variations in orientation. Finally, the template method is 2-D and is unable to select a complex 3-D bone such as the pelvis. Registration perfectly handles these 3 factors and does not introduce such biases. The higher reproducibility of the registration method can be explained by the fact that it provides all observers with identical support in selection. As opposed to the template method, where each observer positions template over allograft image manually, registration automatically overlays the host and allograft images.

Difficulties in classification of category were a limitation of our study. The task was particularly challenging because observers were provided with 2-D slices to estimate areas. Observers reported difficulty in evaluating the 4 criteria, particularly when matching the sacroiliac joint. The visual inspection of the overlay volumes made the selection user-dependent, which explains why selection of the 3 best-matching allografts was not identical between observers. A second limitation was the criteria used to evaluate recipient/allograft matching. The relevance of such criteria is controversial. Grafting of a whole hemipelvis is uncommon, and there are no absolute criteria for allograft selection, because each surgeon has his own preference and each recipient has specific needs. One should keep in mind that the objective of our study was mainly to compare the selection of the 3 best-matching allografts performed using the registration and template methods. To achieve this, it was necessary to qualify the results using a common framework. Our criteria and classification schemes were used with the sole aim of comparing 2 different methods by qualifying allograft/recipient combinations. The criteria and classification used here are irrelevant in a clinical situation.

Our technique was designed to perform volumetric registration. We preferred this to surface- or point-based registration because volumetric registration is reportedly more accurate (West et al. 1999). The mean square metric was chosen because it is quick, reliable, and robust (Popescu et al. 2003). The precision of our registration algorithm, as defined in the literature (Maintz and Viergever 1998), was assessed using the self-matching technique. The trap graft is an idealized input that tests the capability of an algorithm to precisely align 2 identical volumes. Self-matching is the most popular technique to estimate the systematic error of a registration algorithm (Holton-Tainter et al. 1995). Self-matching produced a negligible error since after transformation, 99.98% of bone voxels belonged to the overlapping zone in the overlay volume (Figure 5E). This negligible error was assigned to interpolation error rather than alignment error. Thus, the registration algorithm that we used is precise. Considering that no registration failure was observed, our algorithm is also reliable.

Our registration method is easily accessible to everyone and is now used systematically at our institution for osteochondral or large allograft selection. The technique is difficult to perform when the shape of the recipient's bone has been modified due to bone destruction by a tumor. In that case, under the assumption of body symmetry, the controlateral healthy side is mirrored to replace the affected bone in the registration process. Our findings encourage tissue bankers to leave the standard template method in favor of registration. If the best possible allograft is present in the bone bank stock, then the registration is the most suitable method currently available for detecting it.
